# UV Photoelectron Spectroscopy of Aqueous Solutions

**DOI:** 10.1021/acs.accounts.2c00523

**Published:** 2022-11-28

**Authors:** William G. Fortune, Michael S. Scholz, Helen H. Fielding

**Affiliations:** Department of Chemistry, University College London, 20 Gordon Street, London WC1H 0AJ, United Kingdom

## Abstract

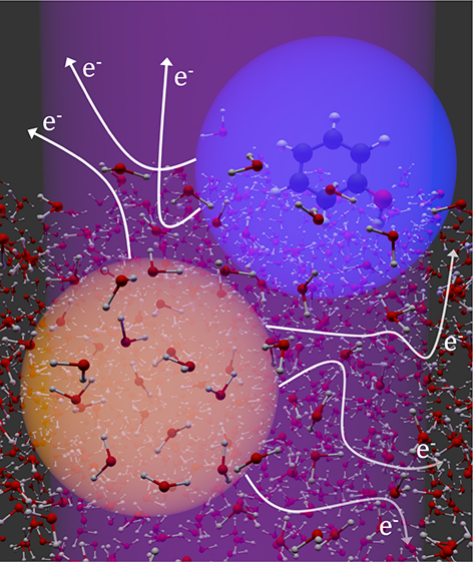

Knowledge of the electronic structure of an aqueous solution is a prerequisite to understanding its chemical and biological reactivity and its response to light. One of the most direct ways of determining electronic structure is to use photoelectron spectroscopy to measure electron binding energies. Initially, photoelectron spectroscopy was restricted to the gas or solid phases due to the requirement for high vacuum to minimize inelastic scattering of the emitted electrons. The introduction of liquid-jets and their combination with intense X-ray sources at synchrotrons in the late 1990s expanded the scope of photoelectron spectroscopy to include liquids. Liquid-jet photoelectron spectroscopy is now an active research field involving a growing number of research groups. A limitation of X-ray photoelectron spectroscopy of aqueous solutions is the requirement to use solutes with reasonably high concentrations in order to obtain photoelectron spectra with adequate signal-to-noise after subtracting the spectrum of water. This has excluded most studies of organic molecules, which tend to be only weakly soluble. A solution to this problem is to use resonance-enhanced photoelectron spectroscopy with ultraviolet (UV) light pulses (*hν* ≲ 6 eV). However, the development of UV liquid-jet photoelectron spectroscopy has been hampered by a lack of quantitative understanding of inelastic scattering of low kinetic energy electrons (≲5 eV) and the impact on spectral lineshapes and positions.

In this Account, we describe the key steps involved in the measurement of UV photoelectron spectra of aqueous solutions: photoionization/detachment, electron transport of low kinetic energy electrons through the conduction band, transmission through the water-vacuum interface, and transport through the spectrometer. We also explain the steps we take to record accurate UV photoelectron spectra of liquids with excellent signal-to-noise. We then describe how we have combined Monte Carlo simulations of electron scattering and spectral inversion with molecular dynamics simulations of depth profiles of organic solutes in aqueous solution to develop an efficient and widely applicable method for retrieving true UV photoelectron spectra of aqueous solutions. The huge potential of our experimental and spectral retrieval methods is illustrated using three examples. The first is a measurement of the vertical detachment energy of the green fluorescent protein chromophore, a sparingly soluble organic anion whose electronic structure underpins its fluorescence and photooxidation properties. The second is a measurement of the vertical ionization energy of liquid water, which has been the subject of discussion since the first X-ray photoelectron spectroscopy measurement in 1997. The third is a UV photoelectron spectroscopy study of the vertical ionization energy of aqueous phenol which demonstrates the possibility of retrieving true photoelectron spectra from measurements with contributions from components with different concentration profiles.

## Key References

- TauO.; HenleyA.; BoichenkoA. N.; KleshchinaN. N.; RileyR.; WangB.; WinningD.; LewinR.; WardJ.; ParkinI. P.; HailesH. C.; BochenkovaA. V.; FieldingH. H.Liquid-microjet photoelectron spectroscopy of the green fluorescent protein chromophore. Nat. Commun.2022, 13, 5073508228210.1038/s41467-022-28155-5PMC8791993.^1^*This UV photoelectron spectroscopy study of the green fluorescent protein chromophore demonstrated the feasibility of measuring accurate electron binding energies of weakly soluble organic chromophores in aqueous solution.*
- ScholzM. S.; FortuneW.; TauO.; FieldingH. H.Accurate vertical ionization energy of water and retrieval of true ultraviolet photoelectron spectra of aqueous solutions. J. Phys. Chem. Lett.2022, 13, 6889–68953586293710.1021/acs.jpclett.2c01768PMC9358712.^2^*Combining Monte Carlo simulations of electron transport in liquid water with spectral retrieval provides an efficient and widely applicable method for obtaining accurate electron binding energies from UV photoelectron spectroscopy measurements of aqueous solutions.*

## 1 Introduction

Photoinitiated chemical reactions are central to a diverse range of processes in nature and technology, from light harvesting and photodynamic therapy to nanoscale machines and electronic devices. Much of our detailed understanding about the electronic structure and relaxation dynamics of the small molecular chromophores that lie at the heart of these processes has been obtained from gas-phase experiments and calculations of isolated chromophores, free from complex interactions with their natural environments.^3−5^ However, electronically excited states can be exquisitely sensitive to their environments, and there is considerable current interest in improving our understanding of how complex environments tune the electronic structure and relaxation dynamics of molecular chromophores.

One of the most direct ways of measuring electronic structure experimentally is to use photoelectron spectroscopy (PES), which records the photoelectron kinetic energy (eKE) distribution following photoionization or photodetachment of an electron. In the independent electron approximation, the photoelectron is removed without any reorganization of the remaining electrons (Koopmans’ picture^6^), and the eKE distribution allows us to determine the electron binding energy (eBE) of the molecular orbital from which the electron was removed, eBE = *hν* – eKE. The spectral profile encodes the role of each vibrational mode of the molecule in the structural relaxation that accompanies the photoionization/detachment process. In the case of solution phase photoelectron spectra, it also contains information about the ultrafast solvent response. Femtosecond time-resolved PES (TRPES) has proved to be a particularly powerful tool for investigating the evolution of electronic structure following photoexcitation.^7−12^ Since photoionization and photodetachment are universal detection methods, TRPES can be used to follow an entire reaction from the moment a photon is absorbed to the formation of products, as long as the photon energy is high enough to remove an electron from the sample.

Until the late 1990s, PES was limited to low vapor pressure samples due to the requirement for high vacuum to minimize scattering of the emitted electrons; however, the development of vacuum liquid microjet technology and its combination with intense X-ray sources at synchrotrons made it possible to probe the electronic structure of volatile liquids, including aqueous solutions.^13^ Since then, liquid microjet PES (LJ-PES) has become an active research field^14^ and liquid-microjets are now also combined with lab-based UV and EUV laser sources.^1,2,14−22^ Unfortunately, a limitation of X-ray LJ-PES is the experimental requirement for solutions with solute concentrations ≳0.2 M in order to obtain a photoelectron spectrum of the solute with adequate signal-to-noise ratio after subtracting the photoelectron spectrum of water (56 M); this has excluded studies of many organic molecules, which tend to be only weakly soluble in water (≲1 mM).^11,23−25^ A solution to this problem is to use resonance-enhanced PES with ultraviolet (UV) light pulses (*hν* ≲ 6 eV).^14,25−33^ Moreover, direct comparison of UV (TR)PES measurements of molecules in both the gas and liquid phases promises to be a particularly straightforward way of unraveling the role of an environment on electronic structure and dynamics.^30^ Nonetheless, until recently,^2,15,34,35^ a lack of concensus on the effect of inelastic scattering on low kinetic energy electrons (eKE ≲ 5 eV) has hampered the development of UV LJ-PES for aqueous solutions. Here, we describe how to account for the effects of inelastic scattering and to retrieve true UV photoelectron spectra from multiphoton measurements of aqueous solutions with excellent signal-to-noise ratio.

## 2 Photoelectron Spectroscopy of Liquids

**Figure 1:**
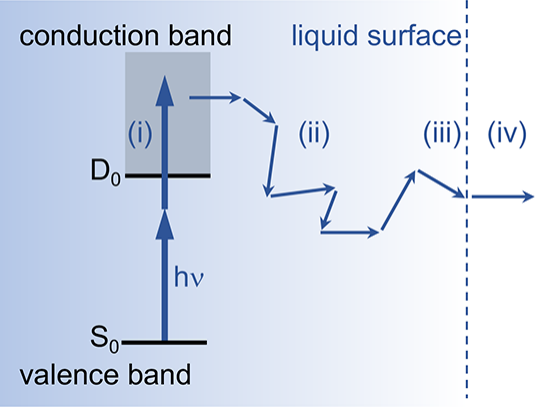
Schematic diagram illustrating multiphoton photoionization/detachment (i) and subsequent electron transport in the conduction band (ii), through the water-vacuum interface (iii), and through the spectrometer (iv). Adapted with permission from ref (1). Copyright 2022 the authors. Published by Springer Nature under a Creative Commons CC BY license.

There are four key steps to obtaining a photoelectron spectrum of an aqueous solution (Figure 1).

(i) Photoionization/detachment generates an initial eKE distribution in the conduction band that is determined by the energy level structure and relaxation dynamics of the species being studied.^36^ The initial spatial distribution of photoelectrons is determined by the focusing conditions and penetration depth of the light source and the depth profile of the species from which the photoelectrons are emitted.

**Figure 2:**
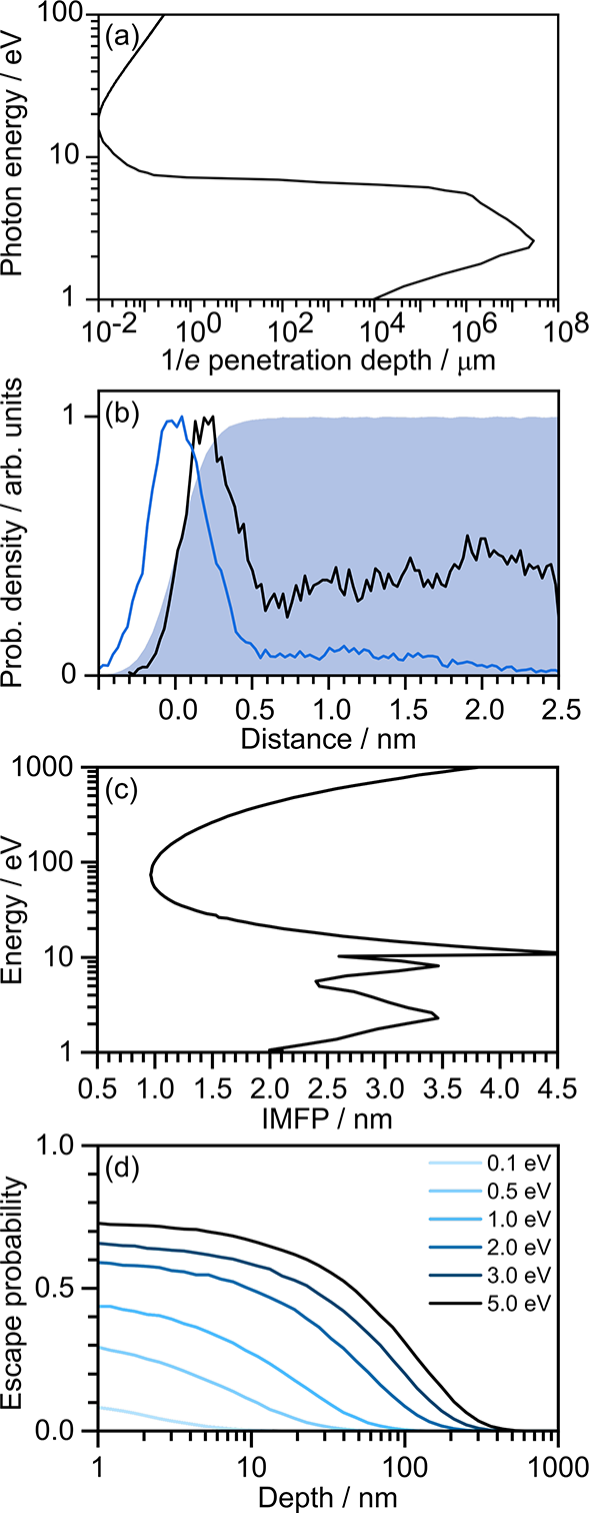
(a) 1/*e* penetration depths for photons in the energy regime 0–100 eV. Data from ref (41). (b) Probability density function for phenolate (black line) and phenol (blue line) in water (blue shading), shown as a function of distance from the liquid–vacuum interface. (c) Inelastic mean free path for electrons in liquid water for a range of electron kinetic energies of ejected electrons. Data from refs (35) (≤10 eV) and (12) (>10 eV). (d) Photoelectron escape probability as a function of eKE and depth below the jet surface calculated using the algorithm from ref (2).

UV light (*hν* ≲ 6 eV) has a penetration depth of several centimeters (Figure 2a), which allows probing of solutes irrespective of their distribution within a liquid-jet (typical diameter ∼20 μm). In the deep UV and EUV regions (8 ≲ *hν* ≲ 20 eV), the penetration depth is ∼100 nm and is thus more sensitive to the surface of a liquid-jet. For *hν* ≳ 20 eV, the penetration depth increases monotonically; this wavelength sensitivity has allowed differences in the electronic structure between bulk and liquid-vacuum interfaces to be studied.^37,38^

**Figure 3:**
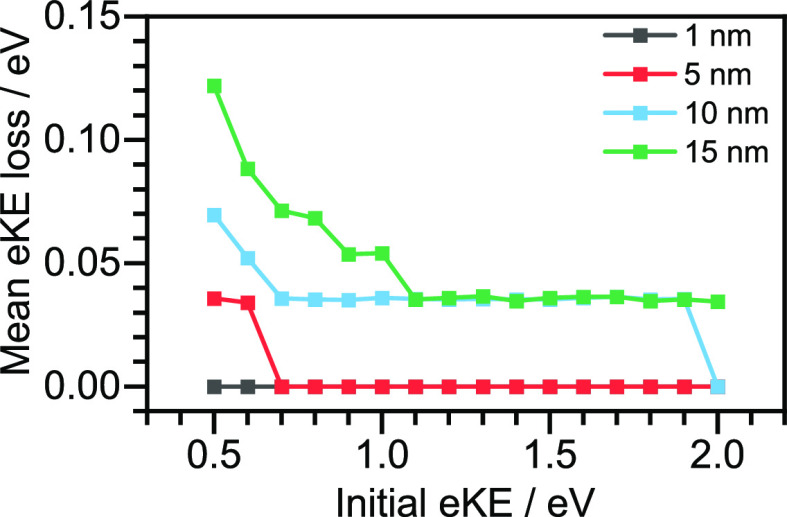
One-dimensional electron scattering simulations, plotted as mean eKE losses as a function of initial eKE for electrons starting at various depths (labeled) in the liquid relative to the surface. Only electrons that emerge successfully from the liquid are included. Adapted with permission from ref (1). Copyright 2022 the authors. Published by Springer Nature under a Creative Commons CC BY license.

Weakly soluble organic solutes tend to have an enhanced surface concentration. Figure 2b shows the solute depth profiles for aqueous solutions of phenol and phenolate, determined using molecular dynamics calculations.^2^ These simulations show that photoionization/detachment of phenol and phenolate will generate initial photoelectron distributions that are predominantly within a nanometer of the surface of the liquid microjet. Enhanced surface concentrations have also been inferred from LJ-PES measurements of deprotonated 4-hydroxybenzylidene-1,2-dimethylimidazolinone (*p*-HBDI^–^),^1^ the green fluorescent protein (GFP) chromophore, and time-resolved LJ-PES measurements of aniline.^32^ Solute distributions depend not only on molecular structure and charge but also on pH and the presence of counterions;^39^ for example, aqueous tetrabutyl ammonium (TBA) enhances the surface concentration of iodide anions compared to less hydrophobic counterions such as sodium.^40^ Variations in solute depth distributions have also allowed differences in the electronic structure between bulk and liquid-vacuum interfaces to be studied.^19^

(ii) After they have been generated, photoelectrons are transported through the conduction band of the aqueous solution to the liquid/vacuum interface. During this process, scattering from liquid water molecules not only reduces the flux (elastic scattering) but also the kinetic energy of the electrons (inelastic scattering). This has the effect of skewing the initial eKE distribution toward lower eKE. For eKE ≲ 20 eV, inelastic electron scattering is dominated by inter- and intramolecular vibrational scattering with energy losses <1 eV. For eKE ≳ 7 eV, electronic inelastic scattering is possible, with energy losses of up to a few eV.^35^ The inelastic mean free path characterizes the distance an electron travels before an inelastic collision. It has a minimum of <1 nm for eKEs in the range 50–100 eV and increases monotonically on either side of this, giving rise to an inverse-bell-shaped curve which is similar for all materials and is referred to as a “universal curve” (Figure 2c). For photoelectrons generated in UV LJ-PES experiments with less than 5 eV eKE, the inelastic mean free path (IMFP) varies within the range 2–3.5 nm, which is greater than the mean of the depth distribution profile of typical organic solutes (Figure 2b). Although the contributions of different inelastic scattering channels vary with energy in a nontrivial way,^35^ this suggests that photoelectrons emitted from weakly soluble organic molecules with an enhanced surface concentration will be essentially free from inelastic scattering. This was supported by preliminary one-dimensional electron scattering simulations of mean eKE loss as a function of initial eKE which have shown that photoelectrons generated within 5 nm of the surface escape with almost no loss of eKE (Figure 3).^1^ In fact, it is clear from Figure 3 that even photoelectrons generated 15 nm from the surface will escape without significant loss of eKE, as proposed by Suzuki and co-workers in 2010.^8^ Nonetheless, UV LJ-PES spectral profiles are still distorted by inelastic scattering, particularly those with photoelectrons that originate from deeper in the liquid-jet and those with lower eKEs.^42,43^

(iii) At the water/vacuum interface, photoelectrons can only escape if their eKE normal to the interface is greater than the electron affinity of water (*V*_0_), i.e. the energy difference between the conduction band and vacuum level. The escape probability, given by *T*(eKE) = 1 – (*V*_0_/(*V*_0_ + eKE))^1/2^,^44^ decreases as eKE decreases, so that no electrons with zero eKE will escape and the probability of low eKE electrons escaping is reduced. The absolute value of *V*_0_ lies in the range 0.1–1 eV. Although the precise value of *V*_0_ has been subject to some discussion,^44−46^ numerical simulations of UV LJ-PES have been found to be fairly insensitive to changing *V*_0_ between 0.1 and 1 eV.^2,46^Figure 2d plots photoelectron escape probability as a function of eKE and depth below the jet surface. For weakly soluble organic molecules within ∼1 nm of the surface of the liquid-jet, almost 60% of photoelectrons with 2 eV eKE will escape, but this falls to less than 10% for photoelectrons with 0.1 eV.

(iv) After they have escaped through the water/vacuum interface, photoelectrons are transported through the photoelectron spectrometer which, for experiments with UV light pulses, is usually a magnetic-bottle (MB) photoelectron spectrometer. Our MB photoelectron spectrometer and experimental procedures have been described in detail in ref (47), although we have made some improvements since then to increase the accuracy of our measurements.^1,2^

**Figure 4:**
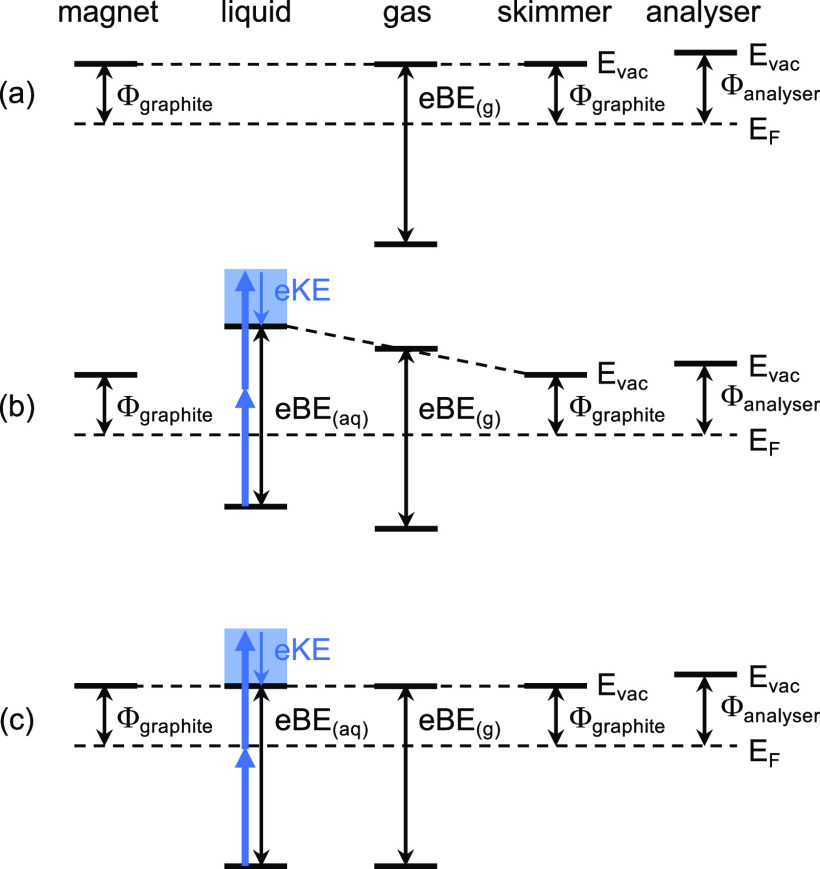
Schematic diagram illustrating the electronic energy levels of the components of a magnetic bottle photoelectron spectrometer in which the magnet, liquid-jet holder, catcher and skimmer are all graphite-coated. Φ_graphite_ and Φ_analyzer_ are work functions, *E*_F_ is the Fermi level and *E*_vac_ is the vacuum level. (a) Without the liquid-jet in place, there is no potential gradient between the magnet and the skimmer. (b) Adding a liquid-jet with a different work function results in a potential gradient that, in this example, accelerates photoelectrons. (c) Adjusting the concentration of electrolytes in the liquid flattens the potential between the magnet and skimmer.

Figure 4 shows the key components of our magnetic bottle spectrometer. When the magnet, liquid-jet holder, liquid-jet catcher and skimmer are all graphite-coated, the vacuum levels are equal and the potential in the interaction region is flat (Figure 4a). Adding a liquid-jet with a different work-function and a streaming potential results in a potential gradient that, in Figure 4b, accelerates the photoelectrons.^13^ The potential gradient can be controlled by adjusting the concentration of electrolyte salt used in the solution, the flow rate, or adding a bias voltage to the solution.^48^ For the measurements we describe in this Account,^1,2^ we have flattened the potential (Figure 4c) by adjusting the concentration of electrolyte salt.

Photoelectron spectra are recorded as a function of electron time-of-flight (ToF), converted to eKE and then corrected for the instrument function and vacuum level offset between the interaction region and the analyzer. We carry out the ToF to eKE calibration using 2 + 1 resonance-enhanced multiphoton ionization (REMPI) of Xe and nonresonant MPI of NO to obtain a series of time-of-flight spectra containing distinct transitions with well-known eKEs. For each NO photoelectron spectrum, the peak intensities of each band are determined relative to the 0–0 vibronic band and their relative variation with eKE is plotted to determine the instrument function. To test whether the potential is flat, we record 2 + 1 REMPI spectra of Xe with the liquid-microjet positioned at a series of distances from the ionization point;^8^ this measurement also allows us to determine the vacuum level offset.^1,2^

## 3 Retrieval of True Photoelectron Spectra

Three approaches have been employed to extract true eKE distributions, *I*_true_(*E*), from measured spectra that have been distorted by inelastic scattering, *I*_meas_(*E*).

(1) Signorell and co-workers employed Monte Carlo simulations to model electron transport, using scattering cross sections determined from photoelectron spectroscopy measurements of liquid droplets.^15,49^ Carrying out repeated Monte Carlo simulations, in a grid search for parameters to fit to UV photoelectron spectra of solvated electrons, allowed the true eBE spectrum of the solvated electron, *e*_(aq)_^–^, to be determined.^15^

(2) Suzuki and co-workers developed a spectral retrieval method based on the assumption that EUV LJ-PES measurements yield the true eKE distribution.^34^ LJ-PES measurements of *e*_(aq)_^–^ were made using both UV and EUV pulses. Inelastic scattering effects were assumed negligible in the EUV spectrum, allowing *I*_true_(*E*) for UV measurements to be determined by shifting the EUV spectrum for a given UV photon energy by *hν*_UV_ – *hν*_EUV_. This *I*_true_(*E*) → *I*_meas_(*E*) linear transformation included, inherently, the effects of inelastic scattering and experimental parameters. Its inverse transformation allowed *I*_true_(*E*) to be determined for UV PES/TRPES measurements of *e*_(aq)_^–^.^34,50,51^ The wide applicability of this approach is slightly limited by the relatively low signals obtained from EUV LJ-PES measurements of weakly soluble solutes and by experimental complexity.

(3) Our group then developed a method combining spectral retrieval and Monte Carlo simulations. The starting point was a basis set of *E*_*z*_ → *S*_*z*_(*E*) transformations, where *E*_*z*_ represented the initial eKE of an electron formed at a distance *z* from the liquid-vacuum interface and *S*_*z*_(*E*) was the eKE distribution leaving the liquid, calculated using Monte Carlo scattering simulations with cross sections determined from photoelectron spectroscopy measurements of liquid droplets.^49^ Following the approach of Suzuki and co-workers,^34^ it was assumed that the true photoelectron distributions were a weighted sum of Gaussian functions, *I*_true_(*E*) = ∑_*i*_*c*_*i*_*G*_*i*_(*E*), where each Gaussian *G*_*i*_(*E*), with weight *c*_*i*_, had its own central eKE and full-width half-maximum (FWHM). Measured UV photoelectron spectra were then fit to a linear combination of *g*_*i*_(*E*), given by *I*_meas_(*E*) = ∑_*i*_*c*_*i*_*g*_*i*_(*E*), where *g*_*i*_(*E*) were the measured eKE profiles representing the effect of distortion by inelastic scattering on the initial Gaussian distributions *G*_*i*_(*E*). The *G*_*i*_(*E*) → *g*_*i*_(*E*) transformations were built “on-the-fly” from the basis set of *E*_*z*_ → *S*_*z*_(*E*) transformations, weighted by the depth profiles of the species from which the photoelectrons were emitted. Guided by the results of our molecular dynamics trajectories of dilute phenol and phenolate aqueous solutions (Figure 2b), we use an exponential function with a mean 0.5 nm into the liquid-jet to describe the concentration profiles of aqueous solutions of organic molecules containing phenol and phenolate building blocks.^2^

**Figure 5:**
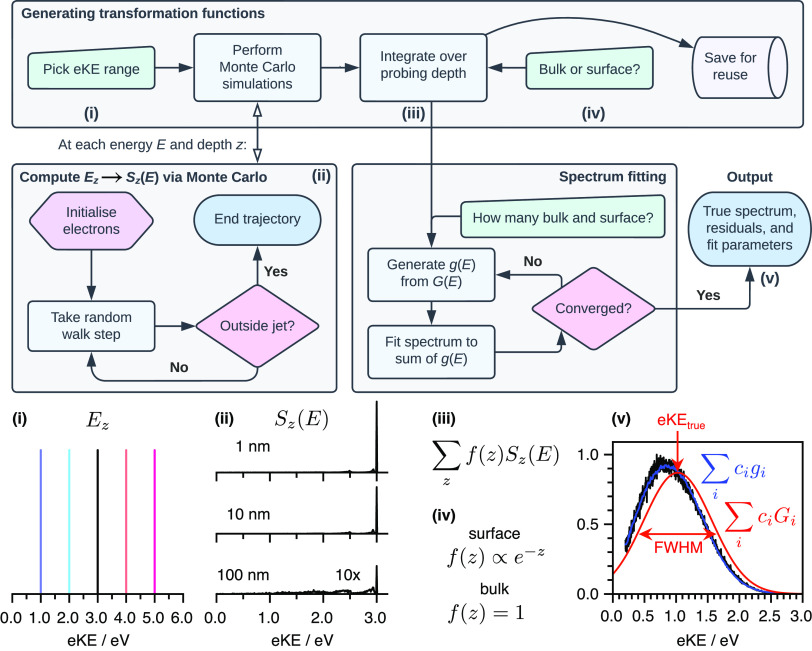
Schematic flowchart of our LJ-PES retrieval algorithm. (i) Having selected the appropriate eKE range based on an experimental measurement, (ii) *E*_*z*_ → *S*_*z*_(*E*) transformation functions are generated using Monte Carlo simulations, (iii) before being integrated over the probing depth *z*, (iv) with each depth weighted by an appropriate function to model the solute or solvent concentration profile. (v) Finally, the experimental spectra are fit to a linear combination of Gaussian functions to which the transformations have been applied, ∑_*i*_*c*_*i*_*G*_*i*_(*E*) → ∑_*i*_*c*_*i*_*g*_*i*_(*E*), to give fit parameters eKE_true_ and FWHM. Panel v is adapted with permission from ref (2). Copyright 2022 American Chemical Society.

A flowchart illustrating our retrieval algorithm is presented in Figure 5. The *E*_*z*_ → *S*_*z*_(*E*) transformation functions take between a few minutes to an hour to compute and are saved to disc for reuse. The fitting procedure takes less than a minute on a laptop computer, including the on-the-fly construction of *G*_*i*_(*E*) → *g*_*i*_(*E*) transformations, and will be straightforward to extend to TRPES by using time-dependent coefficients, *c*_*i*_(*t*): *I*_meas_(*E*, *t*) = ∑_*i*_*c*_*i*_(*t*)*g*_*i*_(*E*) and *I*_true_(*E*, *t*) = ∑_*i*_*c*_*i*_(*t*)*G*_*i*_(*E*).

## 4 Vertical Detachment Energy of the GFP Chromophore in Aqueous Solution

The green fluorescent protein (GFP) emits bright green fluorescence when exposed to blue or UV light. It can be fused to other proteins without impacting their function or their cellular location and has, therefore, been used extensively as a noninvasive tag for following dynamic events in cells.^52^ The chromophore that lies at the heart of the protein has an absorption band centered around 480 nm that is attributed to the first electronically excited singlet state of the deprotonated anionic form of the chromophore (*p*-HBDI^–^). There have been numerous spectroscopic studies of *p*-HBDI^–^ in solution aimed at improving our understanding of the fundamental photophysics of GFP.^53^ The first electronically excited singlet state of *p*-HBDI^–^ is responsible for the bright green fluorescence and its higher lying electronically excited singlet states are believed to be involved in photooxidation processes and in the formation of solvated electrons.^54,55^ In 2001, it was found that the vertical excitation energy (VEE) of the first electronically excited singlet state of *p*-HBDI^–^*in vacuo* was very similar to that of the protein in its anionic form.^56^ This led to the suggestion that the electronic environment of the chromophore in the protein was similar to that of a vacuum and triggered numerous gas-phase studies of the vertical detachment energy (VDE) of *p*-HBDI^–^, the most fundamental property underpinning photooxidation.^57−60^ However, the VDE had not been determined experimentally in any other environment until a recent multiphoton (MP) resonance-enhanced UV LJ-PES study of *p*-HBDI^–^ in aqueous solution.^1^

**Figure 6:**
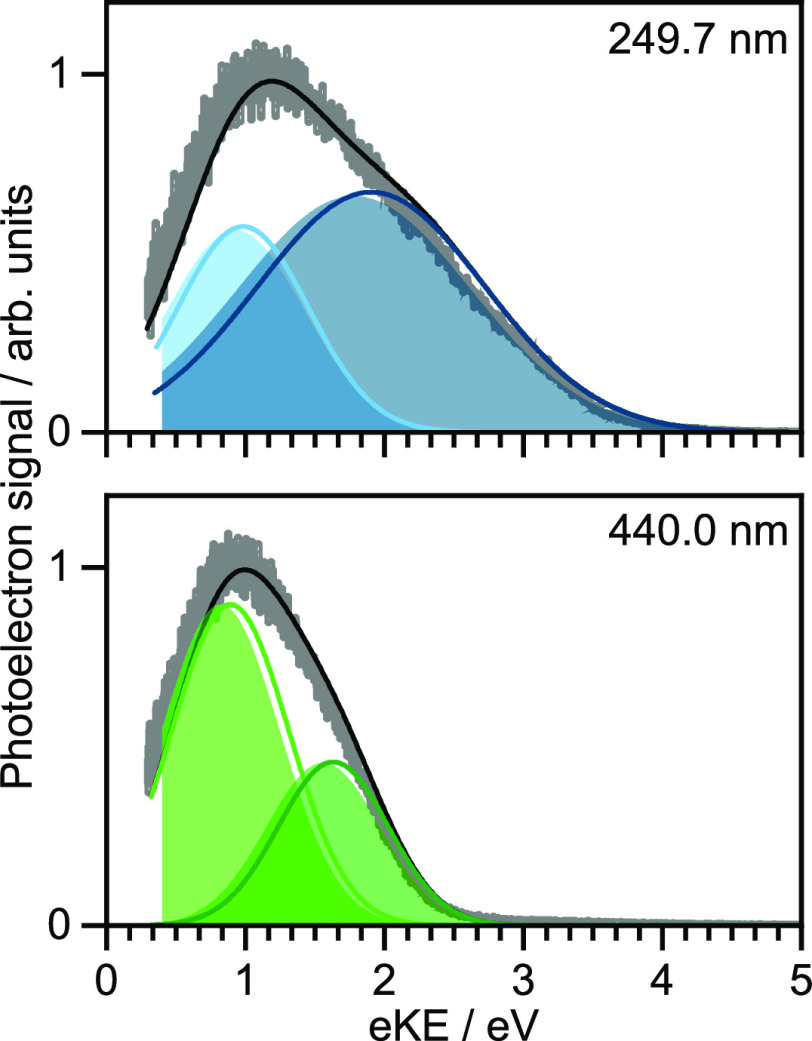
Multiphoton detachment photoelectron spectra of 20 μM aqueous solution of *p*-HBDI^–^ (gray) together with the retrieved *I*_true_(*E*) distributions (black), following photoexcitation at 440 and 249.7 nm, plotted as a function of eKE. Gaussians are fits to the experimental data (filled) and retrieved contributions (lines) and represent S_1_–D_0_ (dark green), S_1_–D_1_ (light green), S_6_–D_1_ (dark blue), and S_5_–D_2_ (light blue) detachment processes. Adapted with permission from ref (1). Copyright 2022 the authors. Published by Springer Nature under a Creative Commons CC BY license.

One-color MP resonance-enhanced photoelectron spectra of 20 μM aqueous *p*-HBDI^–^ using 440 and 249.7 nm are presented in Figure 6. 440 nm is close to the adiabatic excitation energy (AEE) of the S_0_–S_1_ transition and 249.7 nm is resonant with S_0_–S_5_ and S_6_ transitions. The spectra were recorded with a flat potential in the interaction region, corrected for the instrument function of the photoelectron spectrometer and the vacuum-level offset between the aqueous solution and the detector, and best fit to two Gaussians. From high-level quantum chemistry calculations of the electronic structure of the singlet states of the anion and doublet states of the neutral radical and Koopmans’ arguments, it was determined that S_1_ is most likely to detach to D_0_ and D_1_, S_5_ to D_2_, and S_6_ to D_1_.^1^ Assuming that vibrational energy in the resonant intermediate state is conserved during detachment, and ignoring solvent reorganization, VDEs were estimated using VDE(S_0_–D_*i*_) ≈ *mhν* – (*hν* – AEE) – eKE, where *hν* is the photon energy and *m* is the total number of photons involved in the detachment process (*m* = 3 for 440 nm and *m* = 2 for 249.7 nm). S_0_–D_0_ and D_1_ VDEs were determined from the 440 nm photoelectron spectra to be 6.8 ± 0.2 and 7.6 ± 0.2 eV, and the S_0_–D_2_ VDE was determined from the 249.7 nm photoelectron spectrum to be 8.6 ± 0.2 eV. Fitting the measured spectra with Gaussians was justified in terms of *p*-HBDI^–^ being a weakly soluble organic chromophore with an enhanced surface concentration, which results in photoelectrons being emitted essentially free from inelastic scattering. The values obtained this way lie within the errors of refined VDEs that we have since determined using our spectral retrieval software to be 6.8 ± 0.1, 7.5 ± 0.1, and 8.5 ± 0.1 eV for S_0_–D_0_, D_1_, and D_2_ (solid lines in Figure 6).

These experiments were the first reported VDE measurements of any protein chromophore and highlighted the value of multiphoton UV photoelectron spectroscopy for probing the electronic structure of sparingly soluble organic chromophores. Importantly, the first VDE of *p*-HBDI^–^ in aqueous solution (6.8 ± 0.1 eV) was found to be more than double that of the deprotonated chromophore *in vacuo* (2.73 ± 0.01 eV)^60^ and very similar to that in the S65T-GFP protein (7.1 eV).^59^ This contrasts with the VEE of the first electronically excited singlet state of *p*-HBDI^–^, which is very similar in the gas-phase and protein and blue-shifted in aqueous solution.^56^

## 5 Vertical Ionization Energy of Liquid Water

**Figure 7:**
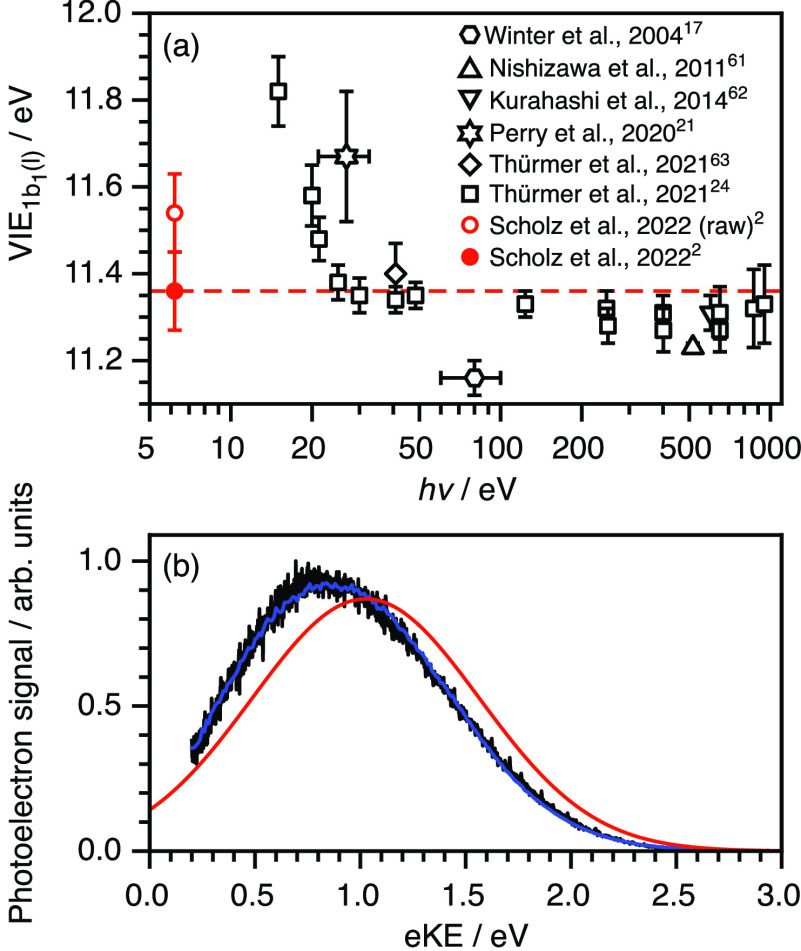
(a) Plot of values of the 1b_1_ vertical ionization energy of liquid water as a function of photon energy. Note that the values measured by Thürmer et al. (open squares)^24^ with *hν* ≲ 20 eV are included to highlight the significant impact of inelastic scattering at these photon energies. (b) Nonresonant two-photon photoelectron spectrum of water (black) following photoionization at 200.2 nm (black) together with the retrieved *I*_true_(*E*) distribution (red). Adapted with permission from ref (2). Copyright 2022 American Chemical Society.

Water is the most important liquid because it is essential for life. Knowledge of its electronic structure is crucial for understanding the interactions between water molecules and with other molecules in aqueous solutions. The vertical ionization energy (VIE) of liquid water is the energy required to remove an electron from its highest occupied molecular orbital, the 1b_1_ molecular orbital. Despite the fact that this fundamental quantity underpins chemical reactivity, there has been a lack of consensus on its value.^2,17,21,24,61−63^ In 1997, Faubel and co-workers made the first measurement of the VIE of liquid water using LJ-PES with a Helium discharge lamp (10.92 eV).^64^ Since then, improvements in our fundamental understanding of streaming potentials,^65^ inelastic scattering processes,^66^ the application of bias voltages and Fermi-level referencing in LJ-PES^24^ has led to refinement of the VIE of liquid water (Figure 7a). The current best estimates have been obtained as an average of several X-ray LJ-PES measurements (11.33 ± 0.03 eV)^24^ and LJ-PES measurements made using a Helium discharge lamp (11.40 ± 0.07 eV).^63^ However, until recently, there had not been any accurate measurements of the VIE of liquid water using UV LJ-PES.^2^

Figure 7b shows a two-photon nonresonant photoelectron spectrum of liquid water, recorded at 200.2 nm with a flat potential in the interaction region, and corrected for the instrument function of the photoelectron spectrometer and the vacuum-level offset between the aqueous solution and the detector. The measured spectrum had a peak maximum of 0.83 ± 0.07 eV, corresponding to a VIE of 11.56 ± 0.09 eV. The retrieved photoelectron spectrum had a peak maximum of 1.03 ± 0.07 eV, corresponding to a VIE of 11.36 ± 0.09 eV, which is in excellent agreement with the current best estimates.^24,63^ The difference between the VIE determined from our raw data and that after accounting for inelastic scattering using our spectral retrieval method is 0.2 eV. For photoelectrons with eKEs around 1 eV (the peak of the retrieved spectrum), the IMFP is around 2 nm (Figure 2c) and photoelectrons generated within a few tens of nanometers still have a reasonable probability of escaping the liquid (Figure 2d) after having undergone several inelastic collisions, although those generated deeper in the liquid will be lost. This explains why the spectrum of liquid water is more distorted and shifted than the spectrum of *p*-HBDI^–^ (Section 4). It is worth noting that VIEs derived from experiments using EUV photon energies around 15 eV can be overestimated by as much as 0.5 eV due to inelastic scattering.^24^

## 6 Vertical Ionization Energy of Aqueous Phenol

Phenol is a ubiquitous biologically relevant structural motif found in numerous biologically relevant chromophores, including the GFP chromophore (Section 4). Its VIE plays an important role in determining the kinetics of charge-transfer processes. The first measurement of the VIE of aqueous phenol was carried out using X-ray LJ-PES (7.8 ± 0.1 eV).^23^ Subsequent measurements of the VIE using resonance-enhanced UV LJ-PES gave values of 7.6 ± 0.1 eV^30^ and 8.0 ± 0.1 eV.^25,67^ Although the measurements gave values that were within experimental error of the X-ray LJ-PES data, they were not in good agreement with one other. The first UV LJ-PES measurements recorded by our group^30^ were analyzed by fitting a single Gaussian to the raw data. In contrast, the photoelectron spectra recorded by Roy et al.^25^ and subsequent measurements by our group^67^ were analyzed by fitting the data to two Gaussians. In their analysis, Roy et al. also included an energy shift to account for inelastic scattering, estimated from photoelectron spectra of *e*_(aq)_^–^ in aqueous solution.^15^

Since these UV LJ-PES measurements, it has become clear that accurate UV LJ-PES measurements can only be carried out with a flat potential in the interaction region and that the vacuum level offset between the interaction region and spectrometer and the instrument function must be taken into account. It is also now clear that energy shifts arising from inelastic scattering of *e*_(aq)_^–^ reported in ref (15). are larger than those expected for phenol, which has an enhanced surface concentration (Figure 2b).^2^

**Figure 8:**
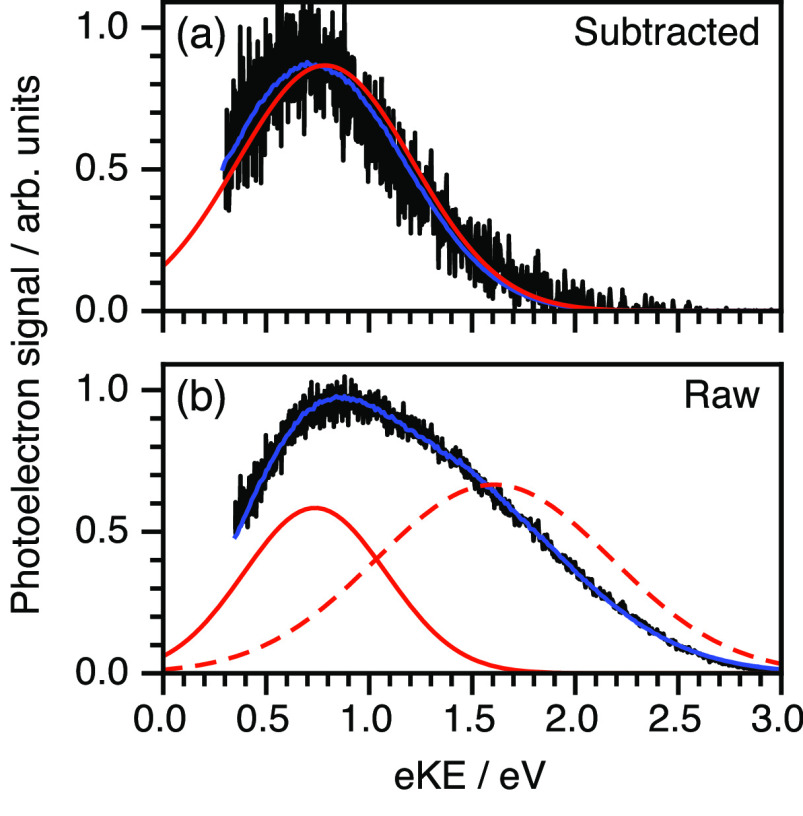
LJ-PES photoelectron spectra of phenol recorded at 290.0 nm. (a) LJ-PES spectrum of phenol after background subtraction and (b) corresponding raw spectrum of phenol including background contribution. The experimental data, fits to the data, and the retrieved initial energy distribution are shown in black, blue, and red, respectively. Adapted with permission from ref (2). Copyright 2022 American Chemical Society.

Figure 8 shows the latest UV LJ-PES measurements of phenol in aqueous solution at 290 nm, just below the onset of the S_0_–S_1_ transition. The data presented in Figure 8a was obtained by subtracting the solvent-only spectrum to isolate the phenol contribution. The retrieved photoelectron spectrum had a peak maximum of 0.79 ± 0.07 eV, corresponding to a VIE of 7.76 ± 0.09, in excellent agreement with the X-ray LJ-PES.^23^ Using the maximum of the raw data at 0.67 ± 0.07 eV gives a VIE of 7.88 ± 0.09 eV, which is 0.12 eV greater than the retrieved value, similar to *p*-HBDI^–^ (Section 4). The spectrum presented in Figure 8b has contributions from both water and phenol and was best fit with two initial Gaussian distributions with different concentration depth profiles. The lower eKE feature was attributed to phenol (with exponential concentration depth profile) and the higher eKE feature was attributed to water (with uniform concentration depth profiles). The lower eKE feature had a peak maximum at 0.74 ± 0.07 eV, corresponding to a VIE of 7.81 ± 0.09 eV, in agreement with the VIE extracted from the background-subtracted spectrum (Figure 8a). The higher eKE feature had a peak maximum of 1.61 ± 0.07 eV, which was attributed to three-photon ionization of water and equated to a VIE of 11.2 ± 0.1 eV. The difference between the VIEs determined from three-photon ionization and two-photon nonresonant ionization at 200.2 nm (Figure 7a) was attributed to a resonance in the absorption spectrum of water at the two photon level.^68−70^ Importantly, this measurement demonstrated that it is possible to retrieve true photoelectron spectra of different components of an aqueous solution with different concentration depth profiles.

## 7 Summary and Outlook

In this Account, we have shown that by combining accurate experimental measurements with our efficient and widely applicable method for retrieving true photoelectron spectra from UV LJ-PES measurements, it is possible to measure electron binding energies with an accuracy that is comparable with state-of-the-art X-ray LJ-PES measurements. The examples chosen to illustrate the power of our methodology included measurements of the VDE of the GFP chromophore and the VIEs of liquid water and aqueous phenol. The measurement of the VDE of the GFP chromophore in aqueous solution led us to realize that inelastic scattering was minimal in UV photoelectron spectra of sparingly soluble organic molecules with an enhanced surface concentration. This work also revealed that the detachment energy was similar to that in the protein, suggesting that the photooxidation properties of the GFP chromophore in aqueous solution may be a good model for those in the protein. The measurement of the VIE of liquid water represented the first accurate UV LJ-PES measurement of this most important liquid and demonstrated that our spectral retrieval method was not restricted to aqueous solutions in which inelastic scattering was minimal. The phenol measurements resolved an uncertainty that had arisen from earlier work about whether spectra recorded using REMPI via the S_1_ state should be fit to one or two Gaussians, highlighting the importance of accurate measurements and spectral retrieval. Moreover, this work also demonstrated that our software was capable of retrieving spectra of components of a solution with different concentration profiles, opening up the exciting possibility of UV LJ-PES becoming a valuable analytical tool.

Our retrieval method is computationally efficient so it can be extended easily to time-resolved measurements. Although there are a range of experimental techniques for probing ultrafast dynamics in aqueous solution, such as transient absorption, LJ-TRPES is particularly appealing because it can be compared directly with analogous TRPES experiments in the gas-phase, thus making it ideal for disentangling the role of an aqueous environment. In summary, UV LJ-PES and its time-resolved variant promise to be powerful tools with the potential to transform our understanding of the electronic structure and relaxation dynamics of aqueous solutions of sparingly soluble organic molecules.
